# Intestinal distension in patients with Crohn’s disease studied by CT and MRI: techniques and review of the literature

**DOI:** 10.1093/bjro/tzae027

**Published:** 2025-07-01

**Authors:** Laura Maria Minordi, Luigi Larosa, Antonio Bevere, Laura Tuzza, Maria Gabriella Brizi, Riccardo Manfredi, Luigi Natale

**Affiliations:** Dipartimento di Diagnostica per Immagini e Radioterapia Oncologica, Fondazione Policlinico Universitario Gemelli IRCCS, 00168, Roma, Italy; Dipartimento di Diagnostica per Immagini e Radioterapia Oncologica, Fondazione Policlinico Universitario Gemelli IRCCS, 00168, Roma, Italy; Dipartimento di Diagnostica per Immagini, Radioterapia Oncologica e Ematologia, Università Cattolica S.Cuore, 00168, Roma, Italy; Dipartimento di Diagnostica per Immagini, Radioterapia Oncologica e Ematologia, Università Cattolica S.Cuore, 00168, Roma, Italy; Dipartimento di Diagnostica per Immagini e Radioterapia Oncologica, Fondazione Policlinico Universitario Gemelli IRCCS, 00168, Roma, Italy; Dipartimento di Diagnostica per Immagini, Radioterapia Oncologica e Ematologia, Università Cattolica S.Cuore, 00168, Roma, Italy; Dipartimento di Diagnostica per Immagini e Radioterapia Oncologica, Fondazione Policlinico Universitario Gemelli IRCCS, 00168, Roma, Italy; Dipartimento di Diagnostica per Immagini, Radioterapia Oncologica e Ematologia, Università Cattolica S.Cuore, 00168, Roma, Italy; Dipartimento di Diagnostica per Immagini e Radioterapia Oncologica, Fondazione Policlinico Universitario Gemelli IRCCS, 00168, Roma, Italy; Dipartimento di Diagnostica per Immagini, Radioterapia Oncologica e Ematologia, Università Cattolica S.Cuore, 00168, Roma, Italy

**Keywords:** MR-enterography, MR-enteroclysis, CT-enterography, CT-enteroclysis, Intestinal distension, hydro-MRI

## Abstract

MRI and CT are routinely performed in patients with Crohn’s disease and allow a panoramic view of the abdominal region, permitting to identify intestinal disease, extraintestinal manifestations, and vascular alteration surrounding the bowel wall. Considering that most errors are related to an insufficient distension of the bowel, the requisite for an adequate MRI or CT study of the intestine is the correct bowel distension in order to have the visualization of the entire bowel. For these reasons, CT and MRI are performed after administration of a contrast medium by mouth (MR-enterography; CT-enterography) or by nasojenunal tube (MR-enteroclysis; CT-enteroclysis). The method of administration of the contrast medium affects the degree of distension of the intestinal loops. In particular, not all small bowel loops are equally distended after administration of the contrast agents by mouth, being the ileum usually better distended than the jejunum. This problem could be solved by using MR-enteroclysis and CT-enteroclysis. In these techniques, contrast medium is administered through the nasojejunal tube, and a better small bowel distension is usually obtained. Even if the study of small bowel disease is the most common indication of MR-enterography or MR-enteroclysis and CT-enterography or CT-enteroclysis, these techniques occasionally may be focused on colon examination. Additionally, water enema may be performed at the end of the MR-enterography (hydro-MRI) to reach optimal colon-rectum distension. In this paper, the authors review the techniques of intestinal distension described in the literature, using some CT and MR examples.

## Introduction

The diagnosis of Crohn’s disease (CD) in adults is difficult and usually requires comprehensive review of the patient’s history, global physical examination, many tests (such as blood tests, stool examination), endoscopy with biopsies and imaging studies, which are fundamental to confirm the diagnosis and exclude other causes. The reference standard for the diagnosis and assessment of the disease activity in patients with CD is the endoscopy. However, it provides information limited only to the mucosal surface and can’t be performed with insurmountable strictures. MRI and CT are routinely performed in CD patients and allow a more panoramic view of the abdominal region, permitting to identify intestinal disease, extraintestinal manifestations, and vascular alteration surrounding the bowel wall. Considering that most errors are related to an insufficient distension of the bowel, the requisite for an adequate MRI or CT study of the intestine is the correct bowel distension to allow the visualization of the entire bowel. For these reasons, CT and MRI are performed after administration of a contrast medium by mouth (MR-enterography [EG], MR-EG; CT-enterography, CT-EG) or by nasojenunal tube (MR-enteroclysis [EC], MR-EC; CT-enteroclysis, CT-EC). In the past few years, MRI advantages have led to a rapid increase in its utilization, even though CT is still the most familiar diagnostic method utilized to study CD patients worldwide. MRI provides a better soft tissue contrast resolution in comparison to CT, ensuring a superior visualization of the bowel wall and its fibrotic and inflammatory alterations.^1^

The method of administration of the contrast medium affects the degree of distension of the intestinal loops. In particular, not all small bowel loops are equally distended after administration of the contrast agents by mouth, being the ileum usually better distended than the jejunum.^2–4^ This problem could be solved by using MR-EC and CT-EC.^2^^,^^5^^,^^6^

Even if the most common indication of MR-EG/EC and CT-EG/EC is studying small bowel disease, occasionally these techniques may be focused on colon examination. The most familiar CT technique for studying the large bowel is colonography-CT.^7^ Another method to distend the colon may be utilizing water as intraluminal contrast media at the end of the CT/MR-EG, also called CT/MR-EG with water enema (CTE/MRE-WE) or hydro-CT/MRI.^8^

In this article, the authors review the techniques of intestinal distension described in the literature, using some CT and MR examples.

## Types of contrast agents

Distension of the bowel in CT and in MR can be achieved utilizing different kinds of contrast agents.

**Figure 1.**
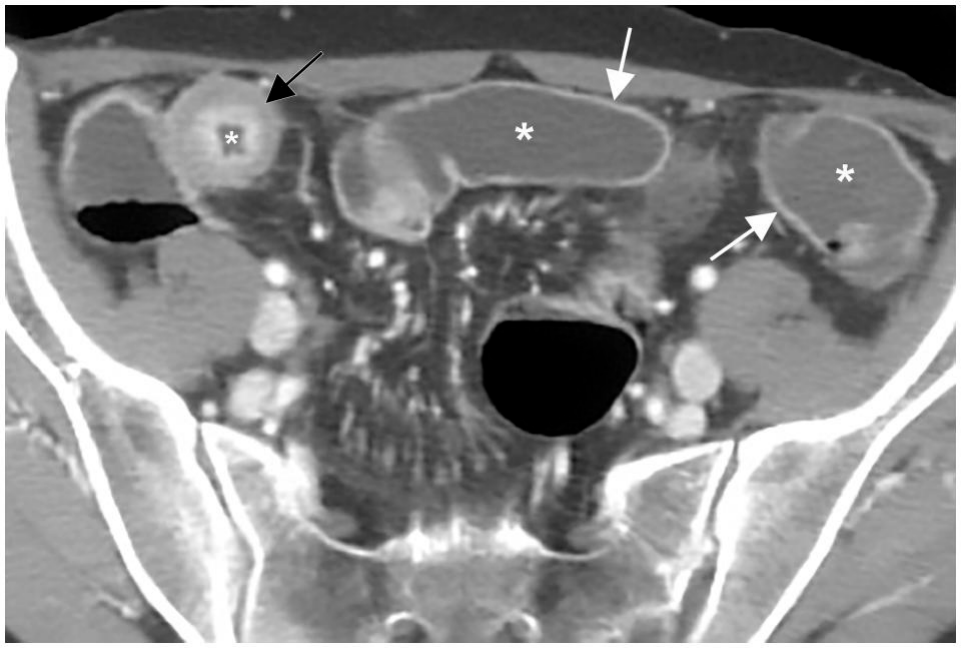
CT-enterography in a patient with known Crohn’s disease. The examination, performed after iodinated contrast medium injection, shows some ileal loops (white asterisks) distended from the oral neutral contrast agent (polyetilen-glycole solution). Hypodensity of the bowel lumen allows better visualization of the intestinal wall and mural enhancement characteristics (white arrows). Marked thickening and stratified contrast enhancement of an ileal loop in the right lower abdominal quadrant are also evident (black arrow).

**Figure 2.**
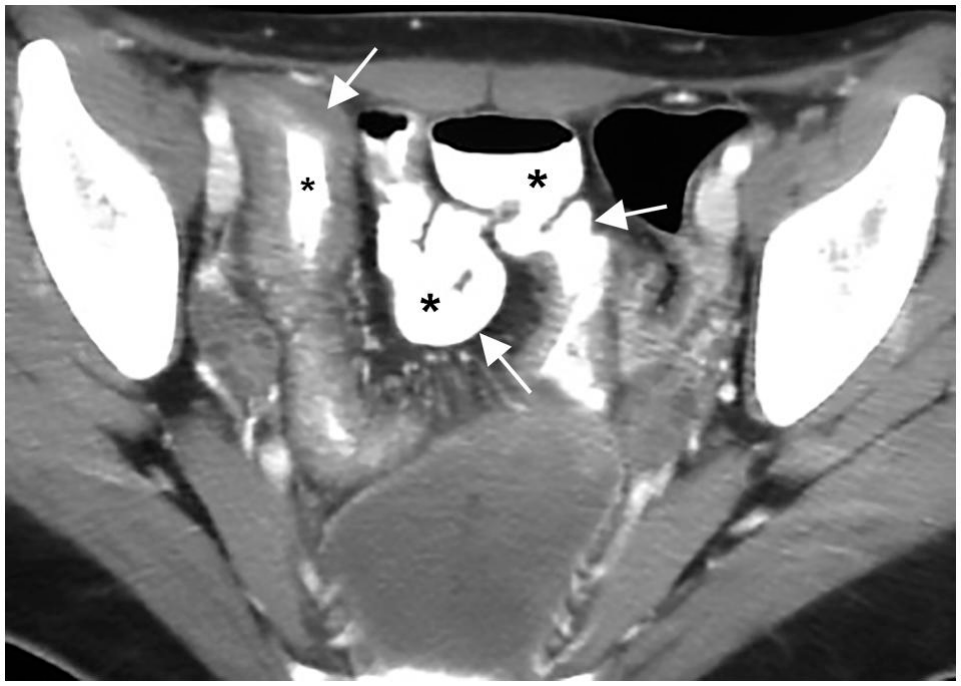
Abdomen CT in a patient with known Crohn’s disease. The examination, performed after iodinated contrast medium injection and orally administration of positive contrast medium (3% water-soluble iodinated solution), shows some pelvic ileal loops, which the lumen is enhanced by positive contrast agent (black asterisks). Hyperdensity of the bowel lumen hides the normal and pathological intestinal walls and the characteristics of their enhancement (white arrows).

In CT, contrast mediums are classified into negative (air, carbon dioxide), neutral (water, polyethylene-glycol solution [PEG], methylcellulose), and positive (2%-3% water-soluble iodinated solution or 1%-2% barium sulphate suspension).^2^^,^^5^^,^^9^ Air may be administered either rectally or orally, but artefacts related to the significant difference between its density and that of the enhanced intestinal wall limit its use. Water is a cheap contrast agent without any toleration issues, which allows proper assessment of the intestinal wall and its enhancement; however, being quickly absorbed and stimulating peristalsis, water is not capable of sufficiently distending the distal jejunum and the ileum. PEG is a well-known contrast agent, also utilized as a preparation for endoscopic studies, which has the advantages of having the same density as water but is not absorbed by the intestine, it has a well-tolerated flavour and no toxicity. Neutral contrast agents allow a better visualization of the small bowel internal wall and the degree of enhancement after intravenous injection of iodinated contrast media (Figure 1). On the other hand, positive contrast agents hide the normal intestinal wall and the characteristics of its enhancement^2^^,^^5^^,^^10^ (Figure 2).

**Figure 3.**
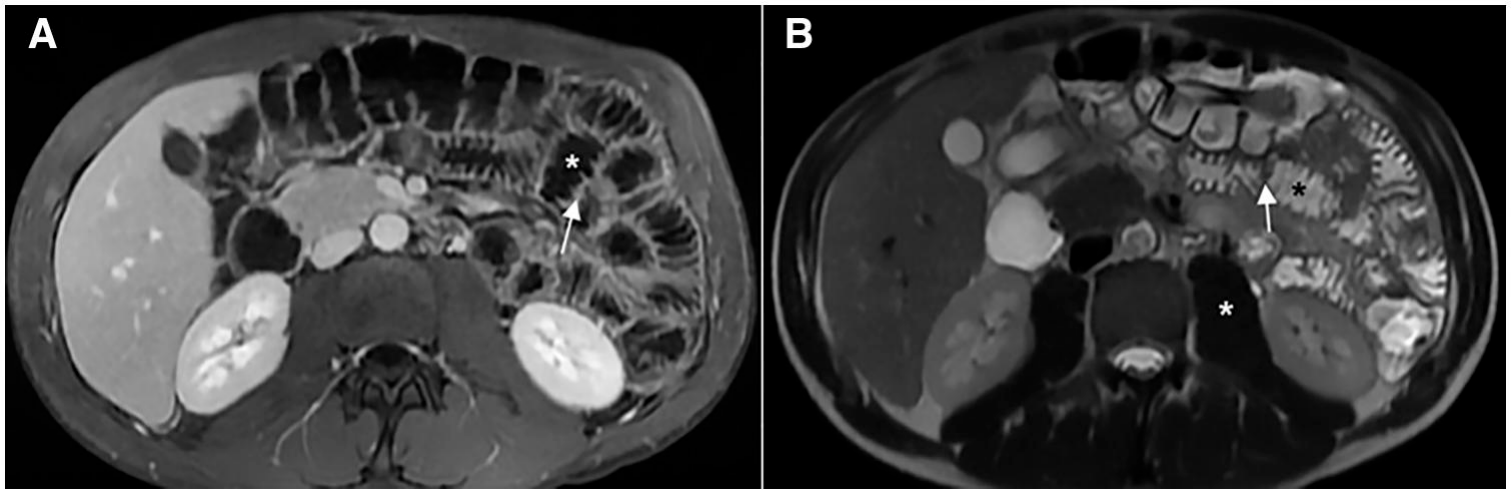
Example of small bowel distension by oral biphasic contrast medium (polyetilen-glycole solution) in MR-enterography. (A) Axial T1-weighted image after gadolinium injection: lumen of jejunal loops is well distended and it shows low signal (white asterisk); the bowel wall (white arrow) shows homogeneous thin enhancement and it is clearly distinguished from lumen. (B) Axial T2-weighted image shows hyperintense signal of lumen of jejunal loops (black asterisk); the bowel wall (white arrow) has an intermediate signal, lower than the lumen (black asterisk) and higher than the psoas muscle (white asterisk).

**Figure 4.**
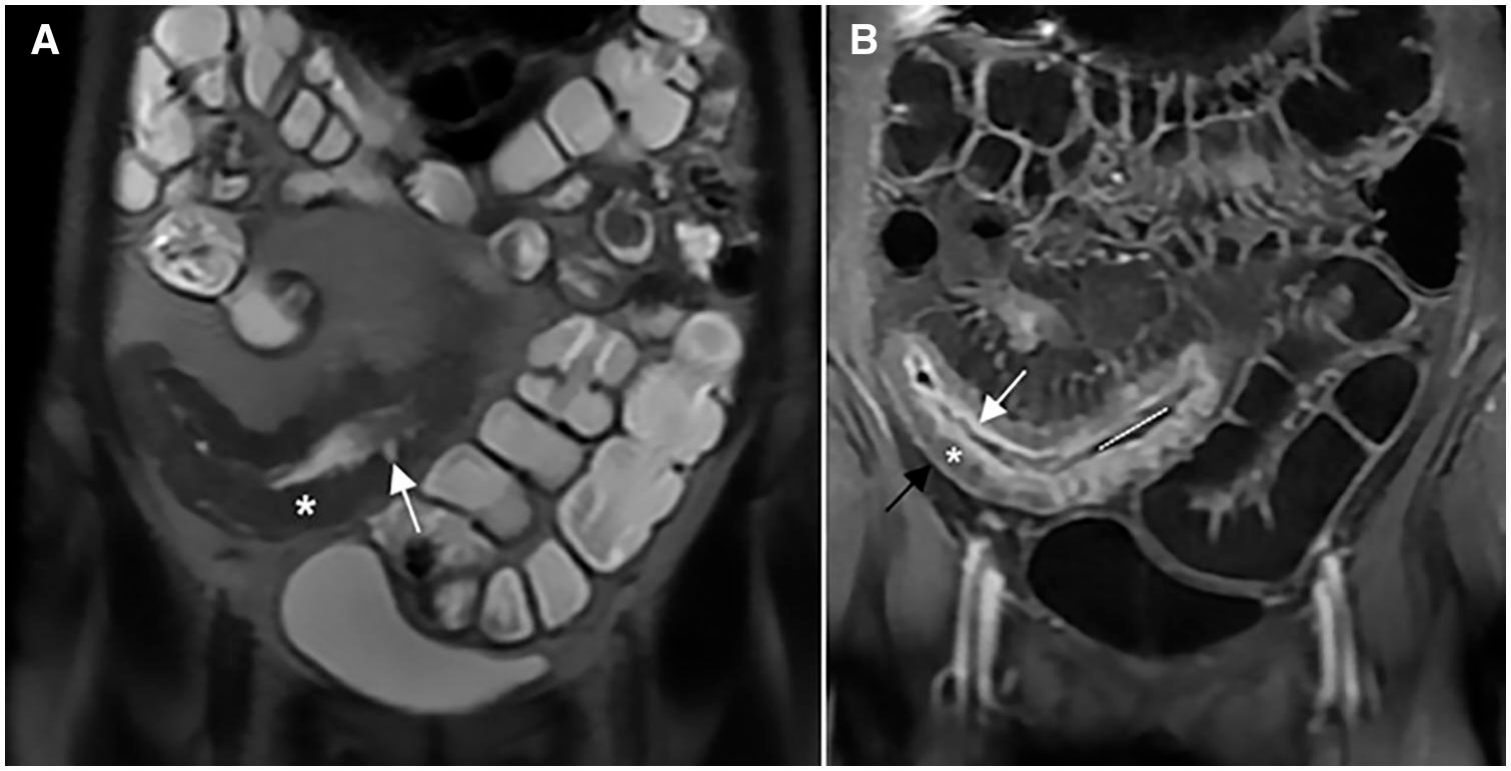
MR-enterography in a patient with known Crohn’s disease: coronal T2-weighted image (A) shows marked thickening of an ileal loop in the right lower abdominal quadrant. Oral biphasic contrast medium (polyetilen-glycole solution) accentuates the signal difference between the lumen and the bowel wall, so it permits to identify wall thickening (white asterisk) and mucosal ulceration (white arrow). The thickness of the small bowel is pathological when it is >3 mm. (B) Coronal T1-weighted image after gadolinium injection shows wall thickening of the ileal loop in the right lower abdominal quadrant with stratified contrast enhancement: the mucosa (white arrow) and the outer layer (the muscle layer and serosa, black arrow) have higher signal due to hyperaemia, while submucosa (asterisk) shows low signal intensity due to oedema. The low signal of the lumen (dashed line) permits us to better appreciate mucosal enhancement. The stratified contrast enhancement is one of the disease activity signs.

In MRI, small bowel distension is typically achieved with a biphasic contrast medium, such as water, polyethylene glycol, mannitol, sorbitol, locust bean gum and diluted barium sulphate.^3^ On T1- and T2-weighted images, these agents are respectively hypo- and hyperintense (Figure 3). Intraluminal abnormalities and trans-mural ulcerations are better identified because of the marked contrast between the bright contrast agents in the lumen and the dark bowel wall on T2-weighted images (Figure 4A). T1-weighted images allow visualizing the degree of enhancement of the bowel wall, thanks to the contrast between the dark bowel lumen and the hyperintense intestinal walls after intravenous injection of contrast agents (Figure 4B). Additionally, a water enema may be performed at the end of the MR-EG (hydro-MRI)^11^ to reach optimal colon-rectum distension in the same examination.

Moreover, a statistically significant correlation between the osmolarity of contrast agents and the degree of bowel distention has been demonstrated with a test level of 0.00001.^12^ The same study also demonstrated a significant linear correlation (level test of 0.001) between the level of osmolarity and the occurrence of adverse events, like diarrhoea, nausea, vomiting, abdominal spasm, and flatulence.

ESGAR/ESPR consensus statement recommends to use mannitol, PEG, sorbitol, or lactulose in CT-EG and MR-EG exams, and mannitol (with or without locust bean gum), PEG, sorbitol, and lactulose in CT-EC or MR-EC.^13^

## Techniques of small bowel distension

Correct bowel distension is requisite for an adequate MR-EG and CT-EG in order to allow the visualization of the entire bowel. Insufficient distension of the small intestine can cause both false-positive and false-negative intestinal findings. In the first case, a little distended loop or an intestinal spasm can be incorrectly interpreted as wall thickening. In the second case, when bowel loops are poorly distended, it is hard to recognize a pathological bowel wall thickening in a not well-distended bowel loop or it may be misinterpreted as a spasm.

There is evidence that good quality CT/MR examinations can be obtained with at least 450 mL of oral contrast agents.^14^ However, Ajaj et al^15^ demonstrated the worse quality of distension when the orally administered volume of contrast agent is <1 L.

Optimal small bowel distension can be achieved through different methods of contrast agent’s administration.

**Figure 5.**
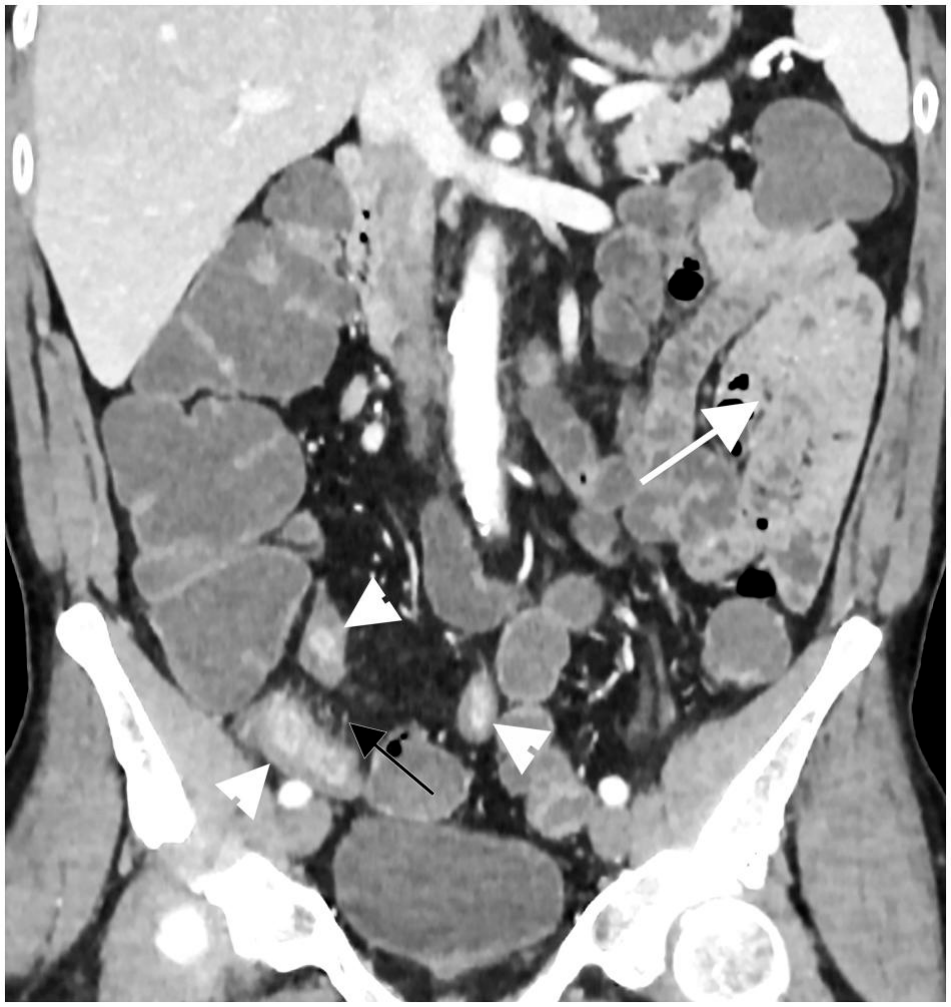
Example of not-good small bowel distension in CT-enterography in a patient with known Crohn’s disease: coronal CT, performed after iodinated contrast medium injection, shows how jejunal loops (white arrow) are not well distended from the oral contrast agents. Some pathological ileal loops are also seen in the right lower abdominal quadrant (white arrowheads) with wall thickening and stratified contrast enhancement; hypervascularity of the involved mesentery is also evident (black arrow).

In MR-EG and CT-EG, contrast agents are generally administered by mouth. Numerous studies regarding oral contrast preparation protocols demonstrated that ileum is typically better distended than jejunum^2–4^^,^^16^ (Figure 5).

The optimal volume of oral contrast ingestion and the time of oral contrast are variable in the literature. ESGAR/ESPR consensus statement^13^ suggests administering 1000-1500 mL in 46-60 min in patients without previous major small bowel resection, a smaller amount if they have undergone bowel resection. Moreover, in 2017, Bekendam et al^17^ made a comparison between small bowel distension after 45 and 60 min of oral contrast preparation, and they noticed an ameliorated small bowel and jejunal distension with the shorter preparation protocol.

**Figure 6.**
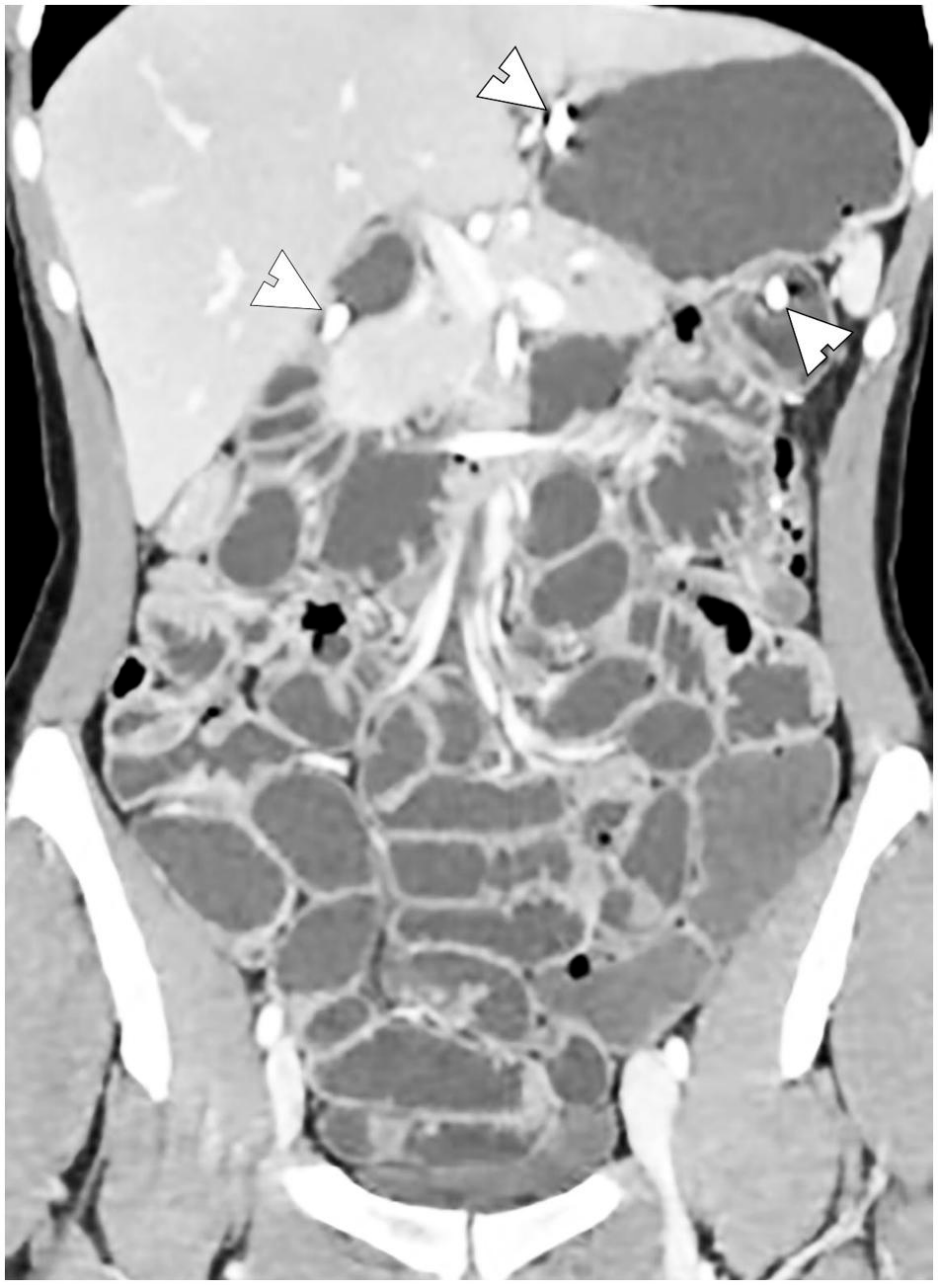
Example of good small bowel distension in CT-Enteroclysis: coronal CT image, acquired after administration of the contrast agent (methylcellulose) through nasojejunal tube and iodinated contrast medium injection, shows adequate homogeneous distension of the whole small bowel. Nasojejunal tube is clearly evident (arrowheads).

In MR-EG and CT-EG, insufficient and nonequally distributed distension of the small bowel, especially jejunum, leads to serious diagnostic issues regarding the distinction of pathological wall thickening and poor distension or spasm. A solution to this inadequate distension may be performing MR or CT-EC, which relies on contrast agent administration through nasojejunal tube by hand or with a peristaltic pump (Figure 6). MR and CT-EC have the main advantage of obtaining an ideal distension of the whole small bowel, comprehending both ileum and jejunum, but at the cost of the higher levels of invasiveness and time.^5^^,^^10^^,^^11^ In fact, some studies have observed that patients complained more discomfort during enteroclysis examinations rather than CT/MR-EG, with a statistically significant difference (*P* < .05). Furthermore CT/MR-EC are time-consuming examinations because it takes about 1 h to prepare patient, including nasojejunal tube placement. The positioning of the tube itself requires higher costs than CT/MR-EG.^2^

At the moment, CT-EG and MR-EG are the diagnostic techniques routinely performed in CD patients and show similar diagnostic accuracy in comparison with enteroclysis techniques in patients with small bowel disease.^6^

CT and MRI techniques for the intestinal distension are described in Table 1.

**Table 1.** CT and MRI techniques.

	Intestinal loops	Administration of contrast medium	Type of contrast medium
CT-EG, MR-EG	Small bowel	By mouth	Neutral, negative, positive
CT-EC, MR-EC	Small bowel	By nasojejunal tube	Neutral, negative, positive
CT-WE (or Hydro-MRI)	Small bowel and colon	By mouth and by enema	Neutral by mouth and water by enema
CT-C	Colon	By enema and by mouth	Negative (air or carbon dioxide) by enema, positive by mouth to tag residual stool

Abbreviations: CT-EG = CT-enterography; MR-EG = MR-enterography; CT-EC = CT-enteroclysis; MR-EC = MR-enteroclysis; CT-WE = CT with water enema; CT-C = CT-Colonography.

The characteristics of the reported papers are in Tables 2 and 3. The authors underline that the number of patients is extremely variable in these studies; in particular, the number of patients examined was ≤50 in 4 papers, over 50 only in 2.

**Table 2.** Small bowel distension: main characteristics of the papers.

Author	Journal and year of publication	N	Study design	Modality of distension	Gold standard	Objective
Minordi et al^2^	*Br J Radiol*, 2011	145	Prospective	Oral and by nasojejunal tube	Surgery	Compare CT-EG and CT-EC in small bowel diseases
Schmidt et al^3^	*Acta Radiol*, 2016	45	Retrospective	Oral and by nasojejunal tube	Biopsy	Comparison of different agents to optimize small bowel distension in MR-EG
Sinha et al^4^	*Indian J Radiol Imaging*, 2013	52	Retrospective	Oral	/	Impact of extended oral preparation on small bowel distension in MR-EG
Maglinte et al^5^	Radiology, 2007	/	Review	By nasojejumal tube	Surgery	Evaluation of CT-EC in comparison to other techniques
Doerfler et al^9^	*Abdom Imaging*, 2013	38	Prospective	Oral	Biopsy	Use of a negative oral contrast material for detecting CD in CT-EC
Engin^10^	*J Comput Assist Tomogr*, 2008	/	Review	By nasojejunal tube	Surgery or Biopsy	Role of CT-EC in small bowel diseases
Young et al^13^	*J Comput Assist Tomogr*, 2008	10	Prospective	Oral	/	Determine small bowel distension, scanning time and side effects of commercially available oral contrast agents in MR-EG
Bekendam et al^14^	*Abdom Radiol*, 2017	50	Retrospective	Oral	/	Compare small bowel distension between a 60-min and a 40-min oral contrast preparation in MR-EG

Abbreviations: CD = Crohn’s disease; CT-EC = CT-enteroclysis; CT-EG = CT-enterography; MR-EG = MR-enterography; *N* = number of patients;.

**Table 3.** Main characteristics of the papers about technique of small bowel distention.

Author	Method	Type (and volume) of contrast medium	Modality of distension	Timing of contrast agent	Splitting of dose
Minordi et al^2^	CT-EG CT-EC	PEG (1.5-2.5 L) MC (1.5-2.5 L)	Oral By nasojejunal tube	45 min before CT scanning Just before the examination	In case of inadequate small bowel distention in the unenhanced scans, administration of further 200-250 mL of methylcellulose or PEG
Schmidt et al^3^	MR-EG	Tylose solution (1.5-1.8 L) Psyllium^a^ (6×3.25 g) LBM^b^ (7.5 mg)	By nasojejunal tube By mouth By mouth	45 min before CT	During the examination At 270, 180, 120, 90, 60, and 30 min prior to the examination 90 min prior to the examination
Sinha et al^4^	MR-EG	3% Mannitol solution (1.2-1.3 L)	Oral	50-55 min before scanning	Divided into 2 aliquots, drink each portion over 25 min + 200 mL just before imaging. Single dose
Maglinte et al^5^	CT-EC	Water (3 L)	By nasojejunal tube	1.5 L infused at 130 mL/min before the scanning and 1.5 L infused at 100-150 mL/min during the examination	/
Doerfler et al^9^	CT-EC	Mucofalk solution (1.5-2 L)	By nasojejunal tube or by mouth	In 60-150 min	/
Engin^10^	CT-EC	0.5% methylcellulose suspension or 4%-15% iodinated contrast medium (2-2.5 L)	By nasojejunal tube	Infusion while the patient is lying on the CT table	/
Young et al^13^	MR-EG	Water (2 L), MC, PEG or LCB (1.3 L) followed by 0.5 L of water by mouth	Oral	From 30 to 90 min before acquisition	Contrast medium was divided into 3 aliquots
Bekendam et al^14^	MR-EG	2% Mannitol (1.6 L)	Oral	60 min before the examination 45 min before examination	Gradually ingest the single dose Contrast agent divided into 3 portions

Abbreviations: CT-EG = CT-enterography; CT-EC = CT-enteroclysis; LBM = locust bean gum; LCB = low-concentration barium; MC = methyilcellulose; MR-EG = MR-enterography; PEG = polyetilen-glycole solution.

a Psyllium: 6 portions of solution containing 3.25 g of psyllium-Mucofalk—dissolved in 250 mL of water.

b LBM: locust bean gum/mannitol (7.5 mg of LBM and 37.5 mg of mannitol dissolved in 1500 mL of water).

## Techniques of colonic distension

**Figure 7.**
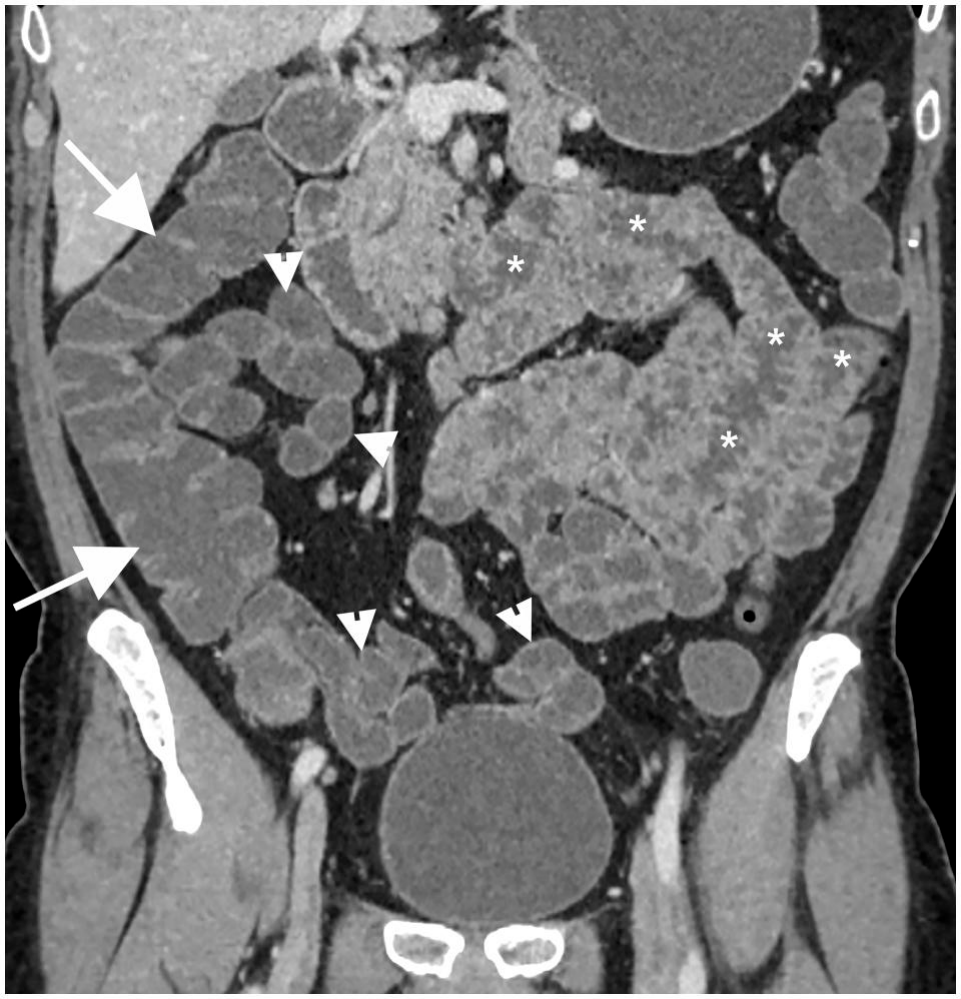
CT-Enterography: neutral contrast agent administered by mouth allows a variable degree of colic distension (white arrows) as seen in this coronal CT image; a good distension of some ileal loops (white arrowheads) has been achieved and acceptable distension of jejunal loops (asterisks) is also evident.

CT- and MR-EG or -EC are performed with contrast agents taken by mouth or by nasojejunal tube in order to obtain small bowel distension. However, contrast medium passes through the ileocecal valve and allows variable degrees of large bowel distension (Figure 7); therefore, pathological findings in the colon can sometimes be identified. However, if the study is aimed at evaluating the colon, other techniques must be employed.

The most familiar CT technique for studying the large bowel is colonography-CT, which relies on the use of air or carbon dioxide.^7^ Furthermore, another method to distend the colon may be utilizing water as intraluminal contrast media at the end of the CT/MR-EG, also called CT/MR-EG with water enema (CTE/MRE-WE) or hydro-CT/MRI. CTE-WE provides a great visualization of the colonic wall (excellent contrast between the hypodense water-filled lumen and the colonic wall) and its parietal enhancement by intravenous contrast media.^8^ Colonic distention is obtained by administering about 1 L of water through a not-traumatic rectal tube, placed when the patient is on the CT table (after oral administration of PEG), before image acquisition. Only a few studies have evaluated the use of CTE-WE in CD.^18^^,^^19^

In 2012, Paparo et al^20^ compared the capability of CTE-WE and CT-EG to detect the prevalence of disease distribution, anastomotic recurrence, behaviour and extraintestinal manifestations in patients with CD. They found that CTE-WE represents a valid diagnostic method to assess disease activity and extraintestinal complications in patients with CD, demonstrating high sensitivity, specificity, and diagnostic accuracy, respectively 89%, 100%, and 92% in patients studied with CTE-WE rather than 77%, 86.5% and 81% with CT-EG.

One more study in 2013 from Paparo et al^8^ focused on the evaluation of the degree of bowel distension obtained by comparing 4 different CT techniques: CT-EC (administration of methylcellulose through a nasojejunal tube), CT-EG (oral administration of PEG solution), CT of the colon with water enema (administration of the water through a rectal enema), CTE-WE (rectal water enema and oral ingestion of neutral contrast material). The grade of large bowel distension, measured both with qualitative and quantitative analysis, was found to be greater and more consistent in CT of the colon with water enema and CTE-WE rather than in CT-EC and CT-EG.

**Figure 8.**
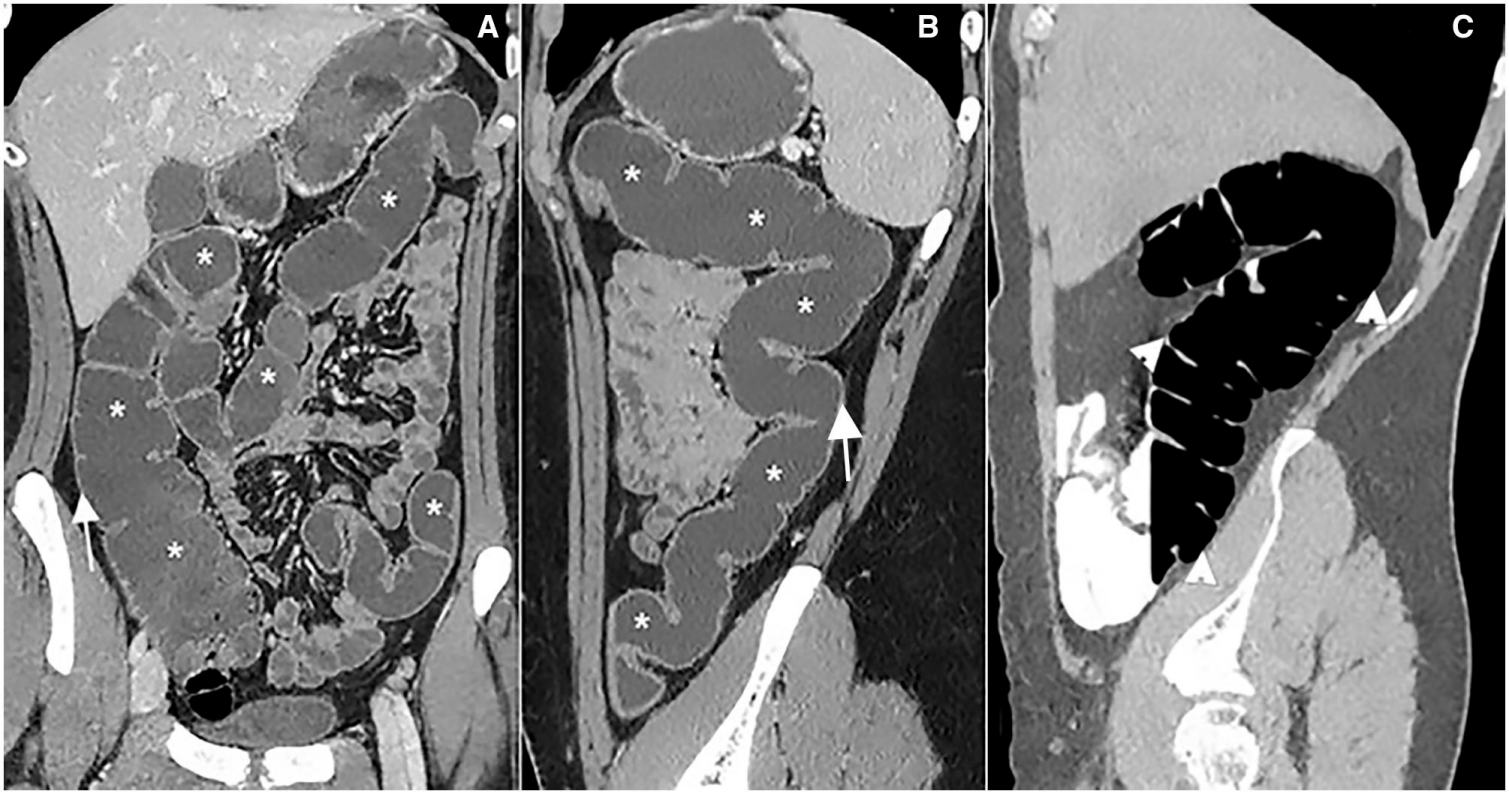
CT with water enema (CT-WE) shows good distension of the large bowel (asterisks) in coronal (A) and sagittal (B) images showing an excellent contrast between the hypodense water-filled lumen and the colonic wall (white arrows in A and B). (C) This technique allows high quality visualization of the entire colonic wall, better than that obtained with CT-colonography, as shown in C (arrowheads).

In a more recent study,^19^ CT-EG and CTE-WE have been compared. In this study, colon distension is notably greater in patients studied with CTE-WE than those studied with CT-EG. The better large bowel distension also correlates to a finer diagnostic accuracy of the CTE-WE compared to CT-EG (in comparison with the reference standard of endoscopy). Regarding patient’s discomfort, even though no significant difference of incidence of nausea and vomiting was found between the 2 groups, presence and degree of abdominal pain in patients undergone to CTE-WE were considerably higher compared to CT-EG. Another reason for discomfort is the positioning of the rectal tube itself, especially in those patients with associated perianal fistulas or anal stenosis. An example of CT with water enema is in Figure 8.

Concerning MR-E, in 2005, Ajaj et al^11^ evaluated the effect of a rectal enema in hydro-MRI to study patients with CD. They found that the additional rectal enema leads to a remarkable better distension and fewer artefacts in both the colon and the terminal ileum (compared to the nonenema group). This results in a higher confidence by the radiologist for the diagnosis of bowel disease, both in the colon and in the ileocecal region.

**Figure 9.**
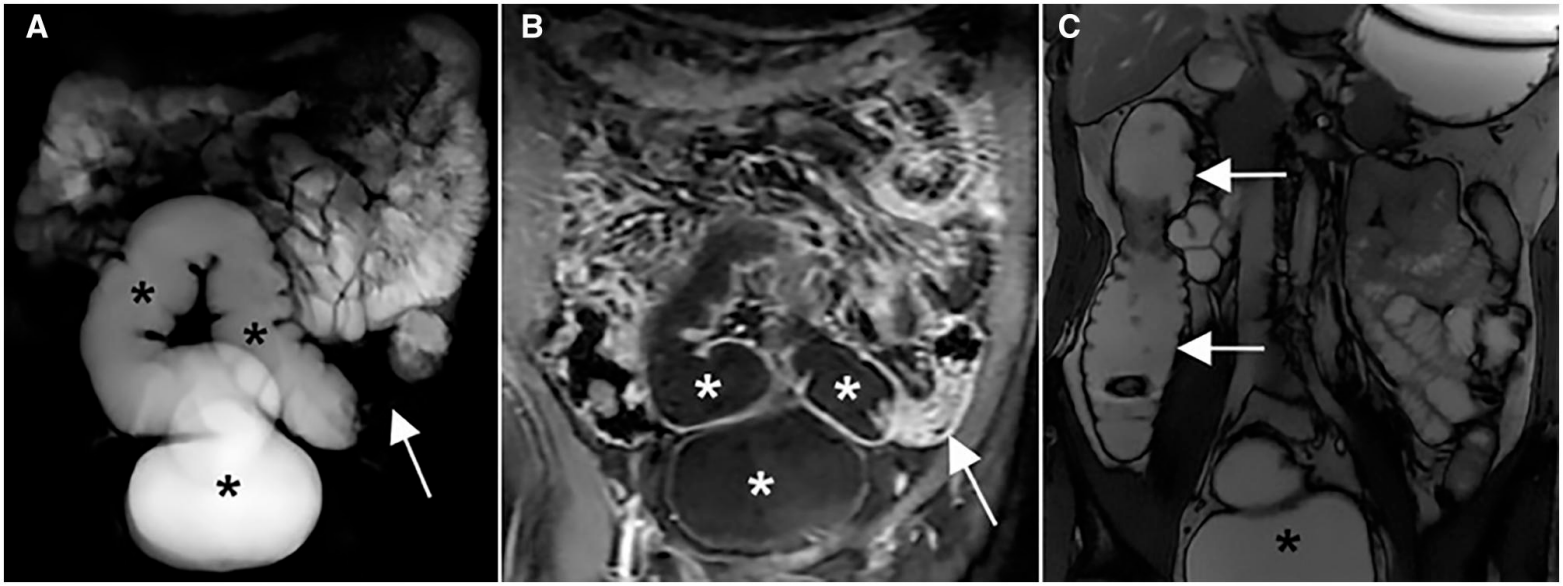
Hydro-MRI in a patient with known Crohn’s disease. Coronal single-shot fast spin-echo (A) and T1-weighted after gadolinium injection (B) images show good distension of rectum and sigmoid colon after rectal enema (asterisks); a stenosis is evident in the proximal sigmoid colon (white arrow); Coronal SSFP Fid + Echo image (C) after rectal enema (black asterisk) demonstrates a good distension of right colon (white arrows).

**Figure 10.**
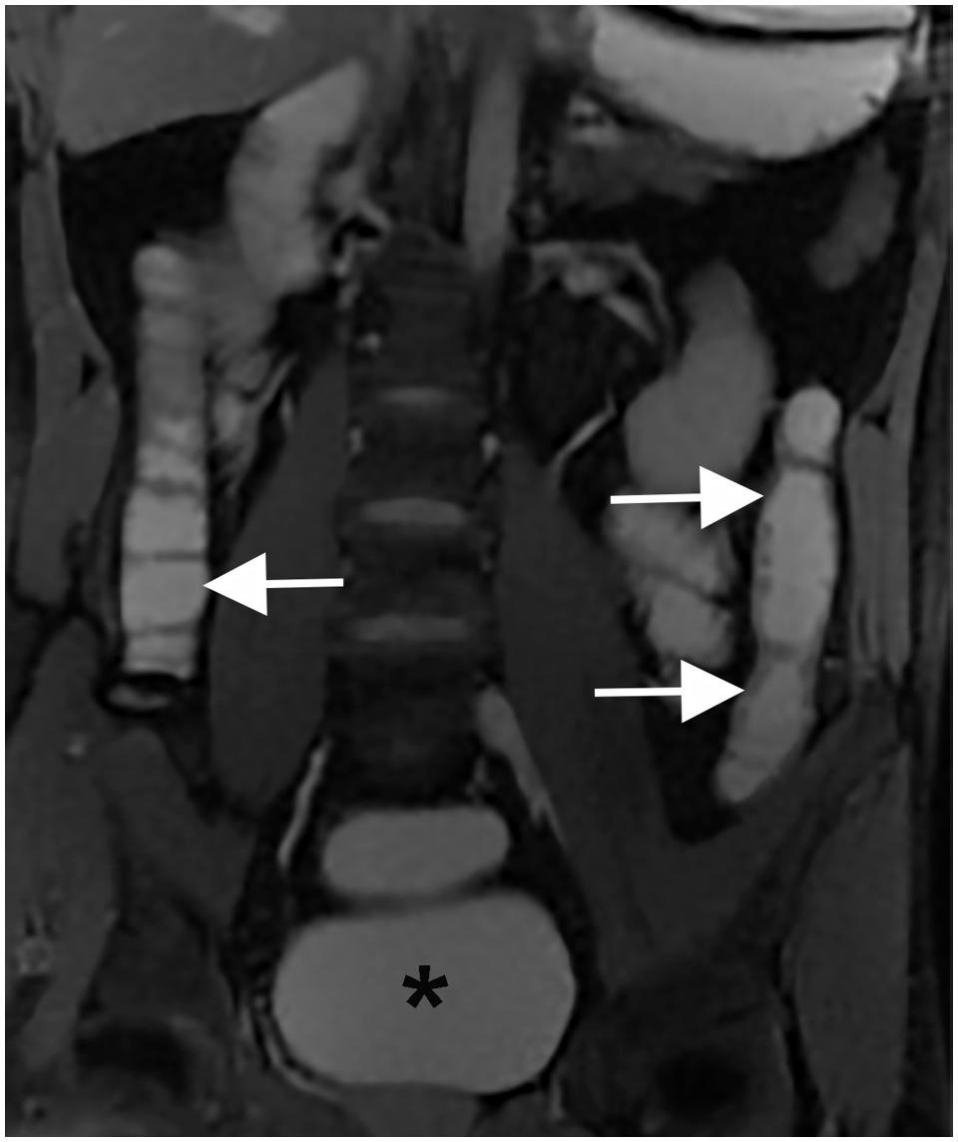
Hydro-MRI in a patient with known Crohn’s disease: coronal T2-weighted image after rectal enema (black asterisk) shows an acceptable distension of ascending and descending colon (white arrows).

Examples of hydro-MRI are in Figures 9 and 10.

Recently, some authors^21^ assessed the stability and diagnostic quality of bowel distension in patients with CD and healthy volunteers, subjected to synchronous colonography and MR-EG. Synchronous colonography and MR-EG obtained diagnostic distension of jejunum in around 80% of patients in both groups. On the other hand, terminal ileum was distended in >94% of patients in both groups, and good and excellent distension was yielded in other bowel segments.

In ESGAR/ESPR consensus statement,^13^ administration of rectal water enema is not recommended before a routine examination. For dedicated colonic evaluation, it is recommended to use water as distension agent by enema where the volume of water rectal enema is based on patient tolerance.

The characteristics of the reported papers are in Tables 4 and 5. The number of patients was extremely variable also in these studies, although higher than those in the articles published on the small intestine distension; in particular, the number of patients was <50 in 2 papers, over 50, and <100 in 3, over 100 in 2.

**Table 4.** Main characteristics of the papers about colonic distention alone or in association with small bowel distension.

Author	Journal, year	*N*	Study design	Method	Distended segments	Gold standard	Objective
Mang et al^7^	*Eur J Radiol*, 2013	/	Review	CT-C	C	Endoscopy	Evaluation of colonic lesions and pitfalls in CT-C
Paparo et al^8^	*Eur J Radiol*, 2013	120	Prospective	CT-EC, CT-EG, CT-WE and CT-WE and CTE-WE	SB and C	/	Compare the grade of bowel distension obtained with 4 different CT techniques dedicated for the examination of the small bowel
Ajaj et al^11^	*J Magn Reson Imaging*, 2005	40	Retrospective	MR-EG and MRE-WE	SB and C	Biopsy	Impact of an additional rectal enema filling in SB hydro-MRI in patients with CD
Paparo et al^15^	*Eur J Radiol*, 2013	51	Retrospective	CTE-WE	SB and C	Endoscopy and biopsy	Diagnostic value of CTE-WE in the assessment of the ileocolic anastomosis in patients with CD
Minordi et al^16^	*Eur J Radiol*, 2016	79	Retrospective	CT-EG and CT-WE	SB and C	Endoscopy	Compare CT-EG vs CTE-WE in patients with CD
Paparo et al^17^	*Abdom Imaging*, 2012	22	Retrospective	CTE-WE	SB and C	Biopsy or surgery	Assess the prevalence of disease distribution, behaviour, anastomotic recurrence and extraintestinal manifestations detected in patient with CD by an original CT technique
Tkalčić et al^18^	*Eur J Radiol*, 2020	164	Retrospective	MR-EG	SB and C	/	Assess diagnostic quality and stability of bowel distension in patients with CD and healthy volunteers subjected to synchronous MR-EG and MR-EC + test the role of water enema and intravenous spasmolytic
Johnson et al^19^	*Emerg Radiol*, 2009	70	Retrospective	CT-EG	C	Endoscopy	Determine if CT-EG is a sensitive and specific method for detecting Crohn’s colitis and ulcerative colitis

Abbreviations: C = colon; CD = Crohn’s disease; CT-C = CT-colonography; CT-EC = CT-enteroclysis; CT-EG = CT-enterography; CT-WE = CT with water enema; CTE-WE = CT-enterography with water enema; MR-EG = MR-enterography; MRE-WE = MR-enterography with water enema; *N* = number of patients; SB = small bowel.

**Table 5.** Main characteristics of the papers about technique of colonic distension alone or in association with small bowel distention.

Author	Method	Type (and volume) of contrast medium	Modality of distension	Timing of contrast agent	Splitting of contrast medium
Mang et al^7^	CT-C	Air	By enema	/	/
Paparo et al^8^	CT-EC CT-EG CT-WE CTE-WE	Methylcellulose (1.8-2 L) PEG (1.5-2 L) Lukewarm tap water (2 L) b + c	By nasojejunal tube By mouth By enema b + c	Flow 60 mL/min Drink in 15-20 min Infused in 3-4 min b + c	/
Ajaj et al^11^	MR-EG MRE-WE	LBG + 2.5% mannitol solution (1.5 L) Lukewarm tap water (0.5-1 L)	By mouth By enema	45 min before the MR examination ND	/
Paparo et al^15^	CTE-WE	PEG (1.5-2 L) + tap water (1.5-2 L)	PEG by mouth and water by enema	PEG 45 min prior to CT + water enema on CT table	/
Minordi et al^16^	CT-EG CT-WE	PEG (2 L) PEG (2 L) and warm water (2 L)	By mouth By mouth and by enema	30-35 min prior to CT a + enema on CT table	equal doses of 100 mL
Paparo et al^17^	CTE-WE	PEG (1-2 L) + tap water (2 L)	PEG by mouth and water by enema	PEG 45 min prior to CT + water enema on CT table	/
Tkalčić et al^18^	MR-EG	0.25% mannitol solution (1-1.5 L)	By mouth	within 1 h	/
Johnson et al^19^	CT-EG	Volumen (1350 mL) + water (250 mL)	By mouth	60, 40, and 20 min before CT scanning	3 × 450 mL of volumen at 60, 40 and 20 min before CT scanning + water immediately before scanning

Abbreviations: CT-EC = CT-enteroclysis; CT-EG = CT-enterography; CT-WE = CT with water enema; CTE-WE = CT-enterography with water enema; CT-C = CT-colonography; LBG = locust bean gum; MR-EG = MR-enterography; MRE-WE = MR-enterography with water enema; ND = not declared; PEG = polyetilen-glycole solution.

## Spasmolytic agent

In general, the use of a spasmolytic agent is fundamental to achieve an optimal and homogeneous bowel distension, to avoid spasms, and decrease abdominal discomfort of the patients.^11^^,^^19^ The most common spasmolytic agents are glucagon or butyl-hyoscine bromide administered intravenously.

In 2009, Johnson et al^22^ assessed the degree of large bowel distension in CT-E studying 70 patients with Crohn’s granulomatous colitis and ulcerative colitis. They performed 47% of the exams with intravenous administration of 0.5 mg of glucagon and the others (53%) without it. Authors found a considerably high percentage of exams to have an inadequate colonic distension for diagnostic purposes in both groups (63% of patients who did not receive glucagon and 66% in the other group), but this evaluation was not made on a per-segment basis.

On the other hand, in another more recent study,^19^ the hypotonic agent was administered in all patients and the assessment of the bowel distension was made considering each segment of the small and large bowel. Distension of colon, sigmoid colon and rectum was significantly higher in patients studied with ECT-WE than those studied with CT-EG, while there was no notable difference for the other bowel segments. Moreover, intravenous administration of the anticholinergic agent (N-butyl-hyoscine bromide) may lower the pain felt by the patients during colon distension in ECT-WE.

Other authors^21^ tested the role of intravenous spasmolytic in patients with CD and healthy volunteers, subjected to synchronous colonography and MR-EG and found that distention of the small bowel was independently and notably improved by using a spasmolytic agent.

The use of the spasmolytic agent in the reported papers is in Table 6.

**Table 6.** Main characteristics of the papers about use of spasmolytic agents.

Author	Method	Type and timing of i.v. spasmolytic agents
Minordi et al^2^	CT-EG, CT-EC	10 mg of i.v. Buscopan when the patient complained abdominal discomfort and 10 mg more just before CT scan
Schmidt et al^3^	MR-EG	10 mg of i.v. Buscopan following the distension phase
Sinha et al^4^	MR-EG	20 mg of i.v. Buscopan when the ileocecal junction was opacified
Maglinte et al^5^	CT-EC	0.3 mg of i.v. glucagon
Doerfler et al^9^	CT-EC	/
Engin^10^	CT-EC	20 mg of i.v. Buscopan or 1 mg of i.v. glucagon
Young et al^13^	MR-EG	/
Bekendam et al^14^	MR-EG	20 mg of i.v. Buscopan or 1 mg of i.v. glucagon before intravenous contrast injection
Mang T et al^7^	CT-C	/
Paparo et al^8^	CT-EC; CT-EG, CT-WE and CTE-WE	Intravenous injection of 2 mL hyoscine-N-butylbromide 20 mg/mL (Buscopan) immediately before CT acquisition
Ajaj et al^11^	MR-EG, MRE-WE	40 mg of Buscopan i.v. immediately before the MR examination or prior to the colonic filling
Paparo et al^15^	CTE-WE	2 mL of hyoscine-N-butylbromide 20 mg/mL (Buscopan) just before CT scanning
Minordi et al^16^	CT-EG, CT-WE	20 mg of Buscopan i.v. just before the CT scan in CT-E or before colonic distension in CT-WE
Paparo et al^17^	CTE-WE	2 mL of hyoscine-N-butylbromide 20 mg/mL (Buscopan) just before CT scanning
Tkalčić et al^18^	MR-EG	20 − 40 mg of scopolamine i.v. prior to T1 weighted sequences
Johnson et al^19^	CT-EG	0.5 mg of i.v. glucagon

Abbreviations: CT-EG = CT-enterography; CT-EC = CT-enteroclysis; CT-C = CT-colonography; CT-WE = CT with water enema; CTE-WE = CT-enterography with water enema; MR-EG = MR-enterography; MRE-WE = MR-enterography with water enema; i.v. = intravenous.

ESGAR/ESPR consensus statement^13^ recommends to use of spasmolytic agents. In particular, the recommended first-line intravenous spasmolytic agent is hyoscine butylbromide and the recommended second line agent is glucagon. The recommended dose is 20 mg for hyoscine butylbromide and 1 mg for glucagon. In MRE, it is also recommended to split the dose before T2W sequences and before contrast-enhanced T1W sequences.

## Drug accelerating gastric motility

Some authors recommend the use of an accelerator of the transit in patients undergoing to CT/MR-EG, in order to induce relaxation of the pylorus, empty the stomach, and prevent nausea and vomiting; metoclopromide is usually used and it is administered intravenously.^9^^,^^16^

On the contrary, Sinha and Rawat^4^ administered an oral suspension of 10 mg of metoclopromide with the first aliquot of mannitol solution; metoclopramide administered by mouth reaches its peak serum concentration at 20-30 min, so it may help in maintaining gastric emptying by overriding the feedback mechanism.

Ajaj et al^11^ administered 100 mg of erythromycin intravenously directly following the ingestion of the first 150 mL of the contrast solution; in the past erythromycin was proved to be a prokinetic agent and proposed to treat gastrointestinal motility disorders.^23^

The use of the transit accelerator in the reported papers is in Table 7.

**Table 7.** Main characteristics of the papers about patient’s position and use of drugs accelerating gastrointestinal motility.

Author	Method	Patient position (aim of the position)	Drugs accelerating gastrointestinal motility
Minordi et al^2^	CT-EG, CT-EC	Supine (ND)	/
Schmidt et al^3^	MR-EG	Supine (ND)	/
Sinha et al^4^	MR-EG	Supine (ND)	/
Maglinte et al^5^	CT-EC	Supine (ND)	/
Doerfler et al^9^	CT-EC	Supine (ND)	10 mg of i.v. metoclopramide before drinking
Engin^10^	CT-EC	Supine (ND)	/
Young et al^13^	MR-EG	Supine (ND)	10 mL of i.v. metoclopramide
Bekendam et al^14^	MR-EG	Supine (ND)	/
Mang et al^7^	CT-C	Supine and prone (ND)	/
Paparo et al^8^	CT-EG, CT-EC, CT-WE and CTE-WE	Supine (ND)	/
Ajaj et al^11^	MR-EG, MRE-WE	Prone (to reduce bowel and respiratory movement, leading to higher image quality)	100 mg erythromycin i.v. following the ingestion of the first 150 mL of the contrast solution
Paparo et al^15^	CTE-WE	Supine (ND)	/
Minordi et al^16^	CT-EG, CT-WE	Supine (ND)	/
Paparo et al^17^	CTE-WE	Supine (ND)	/
Tkalčić et al^18^	MR-EG	Prone (to improve small bowel distension, reduce the number of coronal sections and shorten breath-hold period needed)	/
Johnson et al^19^	CT-EG	Supine (ND)	/

Abbreviations: CT-EG = CT-enterography; CT-EC = CT-enteroclysis; CT-C = CT-colonography; CT-WE = CT with water enema; CTE-WE = CT-enterography with water enema; MR-EG = MR-enterography; MRE-WE = MR-enterography with water enema; i.v. = intravenous; ND = not declared.

## Position

Most of the authors^3–10^^,^^13–19^^,^^21^ report that they used the supine position, but none of them specify the motivation. The prone position was used rather rarely and the authors reported that this position was used in CT for reducing bowel and respiratory movement, leading to higher image quality,^11^ or in MRI to improve small bowel distension, reduce the number of coronal sections, and shorten breath-hold period needed.^21^

The description of the patient’s position in the reported papers is in Table 7.

ESGAR/ESPR consensus statement^13^ does not recommend a particular position.

## Conclusions

Even if small bowel is better distended in CT-EC and MR-EC, at the moment CT-EG and MR-EG are the diagnostic techniques routinely performed in CD patients, showing similar diagnostic accuracy in comparison with CT-EC and MR-EC. CT/MR-EG with water enema ensures a better distension of the large and small bowel, but its limitations include patient’s discomfort and the increased cost and time needed for the procedure, therefore, at present, it is rarely performed in clinical practice.
